# The Association Between Schizophrenia and Cardiovascular Diseases: A Retrospective Cohort Study of Primary Care Routine Data in Germany

**DOI:** 10.3390/brainsci15090974

**Published:** 2025-09-10

**Authors:** Ira Rodemer, Marcel Konrad, Mark Luedde, Karel Kostev

**Affiliations:** 1 Epidemiology, IQVIA, Unterschweinstiege 2-14, 60549 Frankfurt, Germany; 2 Health & Social, FOM University of Applied Sciences for Economics and Management, 60486 Frankfurt am Main, Germany; 3 Medical Clinic I, Cardiology and Angiology, University Hospital of Giessen and Marburg, Campus Giessen, 35392 Giessen, Germany; 4 Department of Cardiology, Christian-Albrechts-University of Kiel, 24118 Kiel, Germany; 5 University Clinic, Philipps-University, 35037 Marburg, Germany

**Keywords:** schizophrenia, cardiovascular disease, heart failure, acute myocardial infarction, chronic ischaemic heart disease, atrial fibrillation and flutter, angina pectoris, incidence

## Abstract

**Background**: This novel study addresses the question of whether schizophrenia is associated with an increased risk of cardiovascular diseases (CVDs) by controlling for metabolic syndrome-related conditions through propensity score matching, using real-world primary care data from Germany. **Methods**: This retrospective cohort study analyzed 12,527 patients aged 18 or older with schizophrenia from 1209 general practices (GPs) in Germany between 2005 and 2023 from the IQVIA Disease Analyzer database. Patients were matched 1:5 with individuals without schizophrenia based on sex, age, index year, consultation frequency, and chronic conditions. CVDs cumulative incidence was assessed using Kaplan–Meier curves and hazard ratios (HRs) were calculated using univariable Cox regression analysis. **Results**: Over a 10-year follow-up, schizophrenia was associated with a higher risk of heart failure (HR: 1.33, 95% CI: 1.20–1.48) and a lower risk of atrial fibrillation and flutter (HR: 0.77, 95% CI: 0.67–0.89). No significant associations were observed for acute myocardial infarction (HR: 0.97, 95% CI: 0.76–1.25), angina pectoris (HR: 0.78, 95% CI: 0.63–0.96), or chronic ischaemic heart disease (HR: 0.91, 95% CI: 0.82–1.02). Stratified analyses showed that schizophrenia was most strongly associated with heart failure in women aged 41–50 years (HR: 3.34, 95% CI: 2.11–5.31), followed by women aged 61–70 years (HR: 1.88, 95% CI: 1.45–2.44) and men aged 51–60 years (HR: 1.81, 95% CI: 1.34–2.45). **Conclusions**: This study highlights significant differences in the 10-year cumulative incidence of CVDs between individuals with and without schizophrenia. While patients with schizophrenia appear less likely to be diagnosed with milder or asymptomatic CVDs, they are at increased risk for severe outcomes. The study’s findings underscore the need for sex-specific and symptom-sensitive public health strategies to improve early detection and prevention of CVDs in patients with schizophrenia.

## 1. Introduction

Cardiovascular diseases (CVDs) remain one of the leading causes of global mortality, accounting for 20.5 million deaths in 2021 [1], and 19.8 million in 2022 [2], nearly one-third of all deaths worldwide.

While overall CVD mortality has declined in high-income countries due to improved prevention and medical care [3], certain vulnerable populations have not benefitted equally from these advancements. One such group is individuals diagnosed with schizophrenia, who face a disproportionately high burden of CVDs and related mortality [4,5]. Neurodegenerative and psychiatric disorders, including schizophrenia, constitute a serious and growing public health challenge worldwide, characterized by complex symptomatology and significant cognitive and social impairments [6]. In this population, CVDs are the leading cause of natural death, which occurs on average ten years earlier than in the general population. Heart disease alone accounts for approximately 25% of these premature deaths. Studies estimate a reduction in life expectancy ranging from 13 to 25 years, largely attributable to cardiovascular conditions [5,7].

A major contributor to this elevated risk is the high prevalence of modifiable cardiovascular risk factors among individuals with schizophrenia, including physical inactivity, obesity, poor diet, metabolic syndrome, type 2 diabetes, dyslipidemia, and hypertension [8,9,10]. Additionally, antipsychotic medications are associated with adverse metabolic effects such as weight gain, insulin resistance, and dyslipidemia, further exacerbating cardiovascular risk [11].

Given the overlap between schizophrenia and metabolic syndrome, it remains crucial to determine whether schizophrenia itself, independent of metabolic syndrome, is associated with an increased risk of CVDs [12]. Addressing this gap, the present study investigates the 10-year incidence of CVDs in patients with schizophrenia using data from general practices (GPs) in Germany between 2005 and 2023 from the IQVIA Disease Analyzer database.

Specifically, the study examines the relationship between schizophrenia and five major cardiovascular outcomes: angina pectoris, acute myocardial infarction, chronic ischaemic heart disease, atrial fibrillation and flutter (AF), and heart failure (HF). To isolate the effect of schizophrenia, the analysis adjusts for metabolic syndrome-related conditions, including essential hypertension, disorders of lipoprotein metabolism and other lipidemias, as well as obesity.

## 2. Database and Methods

### 2.1. Database

The Disease Analyzer (IQVIA) is a comprehensive database that collects anonymized, longitudinal electronic medical record data from general practitioners and specialists across Germany. It contains information on prescriptions, patient demographics, and medical details from approximately 3000 private practices in Germany. These practices included are selected based on several criteria including medical specialty, community size, physicians’ age, and federal state. Reflecting the broader medical landscape in terms of sex, age, and prescription patterns, the database has been validated as representative of general and specialized practices in Germany. More details on the methodology can be found in the scientific literature [13]. The Disease Analyzer has been widely used in various research studies, including those focusing on schizophrenia [14,15,16]. For the present study, data collected between 2005 and 2023 were used.

### 2.2. Study Population

From 1209 GPs in Germany between 2005 and 2023, this study included 12,527 patients diagnosed with schizophrenia (ICD-10: F20) who were at least 18 years old at the index date. A randomly selected visit with a schizophrenia documentation was set as the index date. Patients were required to have at least 12 months of observation time before the index date to ensure no diagnosis of interest was present during the pre-index period, allowing for accurate incidence.

Patients with diagnoses of ischaemic heart disease (ICD-10: I20-I25), atrial fibrillation (ICD-10: I48) or HF (ICD-10: I50) prior to, on the index date, or within three months after the index date were excluded. The three-month exclusion period was applied to reduce the likelihood of including patients with pre-existing but previously undocumented conditions, since diagnoses may not always be recorded at the exact time of disease onset in general practice.

### 2.3. Study Outcomes

The outcomes of the study were CVDs, including angina pectoris (ICD-10: I20), acute myocardial infarction (I21), chronic ischaemic heart disease (I25), AF (I48) and HF (I50), within up to 10 years following the index date. Results were further stratified by sex and age group (18–40 years, 41–50 years, 51–60 years, 61–70 years, >70 years). Each outcome was analyzed separately rather than as a composite endpoint. All stratified analyses were planned a priori.

### 2.4. Statistical Analyses

The baseline characteristics of the study population were categorized by age groups, sex, annual consultation frequency during the follow-up, chronic conditions, and index year (i.e., 2009–2012, 2013–2016, 2017–2020, and 2021–2023). Numerical variables were summarized using mean and standard deviation, while categorical variables were described using absolute numbers and proportions. The standardized mean difference (SMD) was employed to compare the variables included in the propensity matching between the schizophrenia and non-schizophrenia cohorts. An SMD greater than 0.1 indicated a degree of imbalance [17].

To assess the 10-year cumulative incidence of CVDs in patients with schizophrenia and without schizophrenia, Kaplan–Meier curves were generated for both cohorts and compared using the log-rank test. Additionally, the association between schizophrenia and specific CVDs was examined using univariable Cox regression analysis, with models stratified by sex and age groups. Results from the Cox regression models were presented as hazard ratios (HRs) and 95% confidence intervals (CIs). Due to multiple testing, a *p*-value of less than 0.001 was considered statistically significant. All analyses were conducted using SAS version 9.4 (SAS Institute, Cary, NC, USA).

## 3. Results

### 3.1. Basic Characteristics of the Study Sample

The study included a total of 75,162 patients, comprising 12,527 patients with a schizophrenia diagnosis and 62,635 patients without a schizophrenia diagnosis (Table 1). The mean age (SD) of the first cohort was 48.9 years (16.9), while the second cohort had a mean age of 50.0 years (16.9) (SMD = −0.010). In the schizophrenia cohort, 49.3% were female and 50.7% were male, compared to 49.7% and 50.3% in the non-schizophrenia cohort (SMD = −0.007). Patients had an annual consultation frequency during the follow-up of 6.5 (4.6) in the cohort with schizophrenia and of 6.6 (4.6) in the cohort without schizophrenia (SMD = −0.007). Regarding chronic conditions, essential (primary) hypertension was the most frequent comorbidity, affecting 27.1% of patients with schizophrenia and 27.0% of patients without the disease (SMD = −0.002). This was followed by disorders of lipoprotein metabolism and other lipidemias, affecting 16.1% of patients with and 15.9% of those without schizophrenia (SMD = −0.005). Additionally, 10.7% of patients with and 10.4% without schizophrenia were obese (SMD = −0.010). All SMD values were <0.1, demonstrating that the matching procedure resulted in comparable baseline characteristics between patients with and without schizophrenia.

Patients with schizophrenia were matched 1:5 to individuals without schizophrenia using nearest neighbor propensity score matching without replacement. Matching variables included sex, age, index year, consultation frequency, and chronic conditions (essential hypertension [ICD-10: I10], lipid disorders [ICD-10: E78], and obesity [ICD-10: E66]). Covariate balance was evaluated using SMDs, with all values <0.1, indicating adequate balance between cohorts.

### 3.2. 10-Year Cumulative Incidence of CVDs Among Patients with and Without Schizophrenia

**Figure 1:**
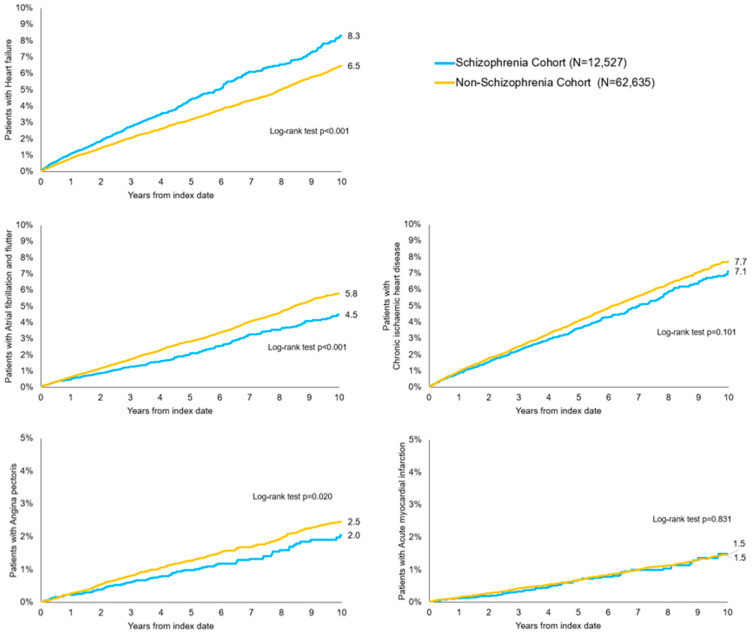
Kaplan–Meier curves for cumulative incidence of cardiovascular diseases (CVDs) in patients with schizophrenia and matched cohorts. Outcomes are shown separately for heart failure, atrial fibrillation and flutter, acute myocardial infarction, angina pectoris, and chronic ischaemic heart disease. Event numbers per outcome are provided in Table 2. Hazard ratios (HRs) with 95% confidence intervals were estimated using univariable Cox regression after propensity score matching.

In the total cohort, significant differences were observed between patients with and without schizophrenia in terms of cardiovascular conditions (Figure 1). Specifically, HF was more frequent among patients with schizophrenia (8.3%) compared to those without schizophrenia (6.5%) (*p* < 0.001). AF as well showed significant differences between the schizophrenia-cohort with 4.5% patients of the schizophrenia-cohort being affected compared to 5.8% patients of the non-schizophrenia (*p* < 0.001). Conversely, angina pectoris and chronic ischaemic heart disease showed a lower cumulative incidence in the schizophrenia cohort, with rates of 2.0% and 7.1%, respectively, compared to 2.5% and 7.7% in the non-schizophrenia cohort. However, these differences did not reach statistical significance (*p* = 0.020 and *p* = 0.101). Acute myocardial infarction was equally distributed in both cohorts with 1.5% (*p* = 0.831).

When stratified by sex, the 10-year cumulative incidence of HF was significantly higher in female patients with schizophrenia (9.4%) compared to those without schizophrenia (7.4%) (*p* < 0.001). Other CVDs did not show significant differences among women. The cumulative incidence of acute myocardial infarction was 1.5% of female patients with schizophrenia and 1.1% in those without (*p* = 0.326). For AF, the rates were 5.0% in the schizophrenia cohort and 6.0% in the non-schizophrenia cohort (*p* = 0.024) among women. Angina pectoris was recorded in 2.2% of female patients with schizophrenia and 2.5% of those without (*p* = 0.050). Chronic ischaemic heart disease was present in 7.2% of female patients with schizophrenia compared to 6.9% of those without (*p* = 0.642).

In male patients, there were no significant differences in the 10-year cumulative incidence of CVDs between the two cohorts. Although 7.2% of male patients with schizophrenia had HF, the difference to 5.5% of men without schizophrenia did not reach statistical significance (*p* = 0.003). Chronic ischaemic heart disease was reported in 7.1% of male patients with schizophrenia and 8.7% of those without the disease (*p* = 0.006). Angina pectoris was present in 1.9% of male patients with schizophrenia and 2.4% of those without (*p* = 0.183). The cumulative incidence of acute myocardial infarction was 1.5% in male patients with schizophrenia and 1.9% in those without (*p* = 0.261). AF was observed in 3.9% of male patients with schizophrenia and 5.6% of those without (*p* = 0.003).

### 3.3. Association of Schizophrenia with Cardiovascular Diagnoses

In the regression analysis, schizophrenia was significantly positively associated with HF in the total population (HR: 1.33; 95% CI: 1.20–1.48), among women (HR: 1.38; 95% CI: 1.21–1.58) but not among men (HR: 1.28; 95% CI: 1.09–1.50) (Table 2). Stratification by age group showed the highest HR in the 41–50-year age group (HR: 2.20, 95% CI: 1.64–2.94), followed by the 51–60-year age group (HR: 1.70, 95% CI: 1.34–2.10) and the 61–70-year age group (HR: 1.62, 95% CI: 1.31–2.00). Other age groups also showed an increased risk of HF in patients with schizophrenia, but the *p*-value of <0.001 was not reached.

When stratified by sex, women aged 41–50 years showed a significant positive association between schizophrenia and HF with the highest HR overall (HR: 3.34, 95% CI: 2.11–5.31), followed by women aged 61–70-years (HR: 1.88, 95% CI: 1.45–2.44). In men, however, only the 51–60-year age group showed a significant increased risk for HF in the schizophrenia cohort (HR: 1.81, 95% CI: 1.34–2.45).

In the total population, schizophrenia was significantly negatively associated with AF (HR: 0.77, 95% CI: 0.67–0.89). However, these results were not replicated in analyses stratified by sex and age groups.

Acute myocardial infarction showed high HR, specifically in women aged 18–40 years (HR: 2.59, 95% CI: 0.62–10.84) but statistical significance was not reached (*p* = 0.193). For chronic ischaemic heart disease and angina pectoris, no notable deviations were found.

## 4. Discussion

This novel study is among the first to investigate the 10-year cumulative incidence of CVDs in patients with schizophrenia using real-world data from general practices in Germany between 2005 and 2023. The results revealed that schizophrenia was associated with a significantly increased risk of HF, with elevated risk observed across both sexes. These findings are consistent with previous studies reporting an elevated HF risk among individuals with schizophrenia [18,19,20,21]. Notably, women aged 41–50 years showed the highest risk in this cohort.

Several mechanisms may underlie the increased HF risk observed in individuals with schizophrenia. An increasing number of studies emphasize shared genetic vulnerabilities, including associations involving the sigma-1 receptor [21] and broader genetic overlaps between schizophrenia and CVDs, specifically HF [22,23].

In addition, individuals with severe mental illness (SMI)—including schizophrenia, bipolar disorder, and major depression—experience cardiovascular mortality rates significantly higher than the general population and exhibit a disproportionately high burden of modifiable cardiovascular risk factors [24,25]. These include but are not limited to poor diet, smoking, and physical inactivity—lifestyle factors that are especially prevalent in this population and contribute to both schizophrenia and CVDs [12]. Consequently, patients with schizophrenia frequently present with comorbid conditions such as hypertension, hyperlipidemia, and type 2 diabetes [8], all of which are well-established risk factors for HF. Although these comorbidities were controlled for in the present analysis, their high prevalence remains a critical concern.

Pharmacological treatment is another important factor in understanding the elevated HF risk in schizophrenia. While essential for reducing psychiatric symptoms and overall mortality, antipsychotic medications and mood stabilizers are associated with adverse metabolic effects, including weight gain, dyslipidemia, and increased risk of type 2 diabetes [11,26,27]. Even though these side effects may vary individually and antipsychotics differ in their metabolic risk profiles, they may nonetheless further exacerbate cardiovascular risk. Clozapine, a commonly used antipsychotic in schizophrenia, is known to be associated with cardiomyopathy [28,29,30], which can lead to HF. Therefore, managing comorbidities and monitoring the side effects of psychiatric treatments is crucial to mitigating cardiovascular risk.

Although this study did not find statistically significant differences in myocardial infarction risk between the cohorts HRs were notably elevated—up to 2.59—in women aged 18–40 years. These non-significant differences might be influenced by the same factors as HF, but due to the lower patient counts, statistical power might not have been sufficient.

Interestingly, a significant negative association was found for AF, with lower cumulative incidence in the schizophrenia cohort (4.5%) compared to the non-schizophrenia cohort (5.8%). However, this association was not consistent across sex- and age-stratified analyses. The observed negative association between schizophrenia and AF may be explained by several factors. Underdiagnosis and underreporting are likely, as cardiovascular conditions are often less intensively screened in patients with schizophrenia, and AF can be asymptomatic or present with non-specific symptoms. Treatment effects may also contribute, since psychotropic medications can alter autonomic regulation and cardiovascular electrophysiology. Finally, biological factors such as differences in autonomic tone, inflammation, or genetic predisposition may play a role. Taken together, these mechanisms could partly explain the lower recorded incidence of AF in this population.

No significant differences were observed for other cardiovascular outcomes, including acute myocardial infarction, chronic ischaemic heart disease, and angina pectoris.

The absence of association for other CVDs observed in this study aligns with previous findings [20]. One possible explanation might be underdiagnosis and underreporting of CVDs in patients with schizophrenia. Prior research has shown that somatic conditions in this population are not only neglected by patients themselves but are also overlooked by people around them and healthcare providers, resulting in disparities in physical health outcomes and limited access to medical care [4,31]. This is consistent with broader evidence indicating that classical modifiable cardiovascular risk factors are poorly screened in patients with SMI. Even when CVDs are identified, individuals with SMI are too rarely treated adequately [26]. Recent studies have shown that despite reductions in cardiovascular risk factors, advances in medical care and declining mortality rates in high-income countries [3], individuals with schizophrenia have not benefitted equally from these improvements and continue to face disproportionately high rates of cardiovascular mortality [32,33]. Given these systemic shortcomings, it is plausible that early-stage or less symptomatic CVDs, such as angina pectoris or chronic ischaemic heart disease, remain undetected, only becoming apparent in more advanced stages like HF. This hypothesis is further supported by the observed lower cumulative incidence of AF in patients with schizophrenia, despite higher HF rates.

Although this study did not find a statistically significant association between schizophrenia and myocardial infarction overall, an elevated HR was observed in younger women (18–40 years), suggesting a potential trend. This trend aligns with genetic studies identifying shared pathways between schizophrenia and myocardial infarction [34], with some evidence pointing to a stronger risk in women [20].

Taken together, the elevated cardiovascular burden in schizophrenia is likely the result of a complex interplay between factors, including genetic predisposition as reported in recent studies, lifestyle behaviors, comorbidities, and treatment-related effects. Addressing this multifactorial risk requires integrated care strategies that go beyond symptom management to include proactive cardiovascular screening and prevention.

## 5. Strengths and Limitations

The findings of this study must be interpreted considering several limitations.

First, the dataset is restricted to outpatient care, which limits the transfer of the findings to inpatient settings. Furthermore, only general practitioners are included as the specialty group documenting both schizophrenia and CVDs diagnoses. As a result, data from psychiatric practices are missing and patients with more severe forms of schizophrenia—often treated in hospitals or psychiatric institutions—may be underrepresented, potentially introducing bias. The absence of hospitalization data may further underestimate serious cardiovascular events such as acute myocardial infarction and heart failure.

Second, diagnoses and comorbidities are based solely on ICD-10 coding, which means that no information is available regarding the severity of schizophrenia or other conditions. In addition, the choice of a random visit with schizophrenia documentation as the index date may introduce bias, as disease stage at inclusion could vary. However, this approach is widely applied in studies using the Disease Analyzer database and was mitigated by requiring at least 12 months of pre-index observation to exclude pre-existing cardiovascular diagnoses.

Third, the database does not include lifestyle-related variables such as tobacco or alcohol use, dietary habits, physical activity, family status, or mortality data, nor does it include data on lipid or sugar profiles of the groups, which could be acting as confounders, potentially influencing the observed associations between schizophrenia and cardiovascular diseases.

Fourth, the dataset lacks information on the context in which initial diagnoses were made. It is possible that general practitioners only document diagnoses received from specialists or hospitals, which may affect the completeness of the data.

Fifth, while the IQVIA Disease Analyzer contains laboratory values (e.g., lipid and glucose profiles), these are only available for a subset of patients and therefore could not be reliably included in this analysis. Consequently, this study relied on diagnoses of metabolic disorders (e.g., hypertension, lipid disorders, obesity) as proxies. This limitation may restrict the ability to fully capture the influence of hyperlipidemia and diabetes as potential confounders. Moreover, the database does not include information on specific cardiac or neuronal biomarkers (e.g., troponin, natriuretic peptides, NSE, NfL, GFAP), which could have strengthened mechanistic interpretations of the observed associations. As such, the study’s findings rely on clinical diagnoses rather than biomarker-supported evidence.

Sixth, HRs were estimated using univariable Cox regression after propensity score matching rather than a multivariable model. Although matching balances observed covariates, any residual confounding due to unmeasured or insufficiently balanced factors remains possible. Future studies using doubly robust methods or multivariable adjustments post-matching could mitigate this limitation. Another methodological consideration is that all standardized mean differences after matching were <0.01, which could raise concerns about potential overmatching. However, the matching procedure was restricted to demographic factors and selected cardiovascular risk factors chosen a priori (sex, age, index year, consultation frequency, hypertension, lipid disorders, obesity). While this ensured excellent balance and reduced confounding, it may also have attenuated some natural variability between groups. Unmeasured confounding therefore cannot be fully excluded.

Seventh, the exclusion of patients diagnosed with heart disease within three months after the index date may have led to the omission of some true incident cases. This design choice was made to minimize misclassification of pre-existing but undocumented conditions as incident cases, but it may have introduced a conservative bias.

Finally, information on psychiatric therapies is not available, as these treatments are typically prescribed by psychiatrists and are not recorded in general practitioner documentation. This limitation is particularly relevant given evidence of sex-specific differences in the prescription of antipsychotics [35], which may help explain some of the sex disparities observed in the study’s findings.

Despite the limitations the IQVIA Disease Analyzer holds, there are several notable strengths. Its broad and diverse patient population makes it a representative source for routing outpatient care in Germany. The longitudinal structure of the data allows for long-term follow-up, allowing researchers to track disease progression and treatment patterns over time. It therefore provides important clinical and epidemiological relevance to the findings.

## 6. Conclusions

This study highlights the elevated cumulative incidence of cardiovascular disease among individuals with schizophrenia. It is essential to raise awareness—not only among affected individuals but also among their social environment and healthcare providers—that this population might not only be at increased risk for cardiovascular diseases, in general, but also less likely to be diagnosed and receive adequate treatment. As a result, severe cardiovascular outcomes such as HF may occur more frequently and could potentially be prevented through routine cardiovascular screening in this vulnerable population. To address these disparities, targeted prevention strategies and integrated care models are needed. Moreover, future research should further explore the underlying mechanisms driving the associations observed in this study, including genetic, behavioral, and systemic factors.

## Figures and Tables

**Table 1:** Baseline characteristics of the study sample (after 1:5 propensity score matching).

	Patients with Schizophrenia (N = 12,527)	Patients Without Schizophrenia (N = 62,635)	Standardized Mean Difference ^a^
**Sex**			
Female	6.180 (49.3)	31.130 (49.7)	
Male	6.347 (50.7)	31.505 (50.3)	−0.007
**Age (in years)**			
Mean (SD)	49.8 (16.9)	50.0 (16.9)	
18–40	4.067 (32.5)	20.091 (32.1)	−0.010
41–50	2.481 (18.8)	12.091 (19.3)	
51–60	2.823 (21.4)	13.657 (21.8)	
61–70	1.864 (14.1)	8.706 (13.9)	
>70	1.923 (14.6)	8.090 (12.9)	
**Annual consultations during the follow-up, mean (SD)**	6.5 (4.6)	6.6 (4.6)	−0.007
**Chronic conditions**			
Essential (primary) hypertension	3.398 (27.1)	16.935 (27.0)	−0.002
Disorders of lipoprotein metabolism and other lipidemias	2.010 (16.1)	9.943 (15.9)	−0.005
Obesity	1.334 (10.7)	6.482 (10.4)	−0.010
**Index year**			
2005–2008	1.047 (8.4)	5.209 (8.3)	−0.002
2009–2012	1.542 (12.3)	7.653 (12.2)	
2013–2016	2.462 (19.7)	12.356 (19.7)	
2017–2020	3.531 (28.2)	17.646 (28.2)	
2021–2023	3.945 (31.5)	19.771 (31.6)	

Proportions of patients in N, % given, unless otherwise indicated. SD: standard deviation. ^a^ The standardized mean difference is commonly used to compare the distribution of covariates included in the propensity score matching, with differences greater than 0.1 indicating a degree of imbalance. Regarding age and index year, the standardized mean difference was computed for the continuous variable only.

**Table 2:** Association between schizophrenia and CVDs in patients followed in GPs in Germany.

	Acute Myocardial Infarction		Chronic Ischaemic Heart Disease		AF		Heart Failure		Angina Pectoris	
Age Group	HR (95% CI)	*p*-Value ^1^	HR (95% CI)	*p*-Value ^1^	HR (95% CI)	*p*-Value ^1^	HR (95% CI)	*p*-Value ^1^	HR (95% CI)	*p*-Value ^1^
**Total**	0.97 (0.76–1.25)	0.832	0.91 (0.82–1.02)	0.102	**0.77** **(0.67–0.89)**	**0.0002**	**1.33** **(1.20–1.48)**	**<** **0** **.0001**	0.78 (0.63–0.96)	0.021
**≤40**	1.85 (0.76–4.49)	0.177	1.22 (0.80–1.88)	0.357	1.15 (0.65–2.05)	0.625	2.01 (1.30–3.12)	0.002	1.01 (0.58–1.74)	0.982
**41–50**	1.28 (0.72–2.28)	0.401	1.28 (0.99–1.66)	0.059	1.40 (0.95–2.07)	0.085	**2.20** **(1.64–2.94)**	**<** **0** **.0001**	0.66 (0.41–1.09)	0.106
**51–60**	0.79 (0.48–1.30)	0.358	1.00 (0.82–1.23)	0.978	0.93 (0.69–1.27)	0.663	**1.70** **(1.34–2.10)**	**<** **0** **.0001**	0.77 (0.52–1.15)	0.202
**61–70**	1.28 (0.79–2.06)	0.313	0.89 (0.72–1.11)	0.314	0.90 (0.70–1.17)	0.427	**1.62** **(1.31–2.00)**	**<** **0** **.0001**	0.65 (0.40–1.07)	0.088
**>70**	0.77 (0.41–1.44)	0.407	0.83 (0.65–1.04)	0.109	0.68 (0.53–0.86)	0.001	1.11 (0.93–1.33)	0.251	1.21 (0.76–1.91)	0.418
**Women**										
**Total**	1.20 (0.83–1.75)	0.326	1.04 (0.89–1.21)	0.638	0.81 (0.68–0.97)	0.024	**1.38** **(1.21–1.58)**	**<** **0** **.0001**	0.75 (0.56–1.00)	0.051
**≤40**	2.59 (0.62–10.84)	0.193	1.37 (0.72–2.63)	0.342	0.87 (0.30–2.54)	0.797	3.03 (1.53–6.00)	0.002	0.93 (0.32–2.73)	0.890
**41–50**	1.30 (0.42–4.01)	0.643	1.48 (0.99–2.22)	0.055	1.52 (0.82–2.80)	0.182	**3.34** **(2.11–5.31)**	**<** **0** **.0001**	0.43 (0.19–1.00)	0.049
**51–60**	1.11 (0.51–2.40)	0.796	1.16 (0.85–1.59)	0.343	1.24 (0.80–1.91)	0.330	1.60 (1.18–2.16)	0.003	0.73 (0.42–1.29)	0.278
**61–70**	1.68 (0.85–3.32)	0.134	1.17 (0.88–1.55)	0.285	1.05 (0.74–1.47)	0.797	**1.88** **(1.45–2.44)**	**<** **0** **.0001**	0.77 (0.43–1.38)	0.382
**>70**	0.99 (0.49–2.01)	0.975	0.83 (0.62–1.12)	0.231	0.67 (0.50–0.89)	0.006	1.07 (0.86–1.33)	0.541	1.15 (0.65–2.03)	0.640
**Men**										
**Total**	0.82 (0.58–1.16)	0.262	0.81 (0.69–0.94)	0.007	0.73 (0.59–0.90)	0.003	1.28 (1.09–1.50)	0.003	0.82 (0.61–1.10)	0.184
**≤40**	1.51 (0.48–4.74)	0.484	1.13 (0.64–2.01)	0.673	1.32 (0.67–2.60)	0.430	1.55 (0.87–2.76)	0.137	1.03 (0.55–1.94)	0.926
**41–50**	1.31 (0.67–2.56)	0.435	1.18 (0.84–1.66)	0.338	1.35 (0.82–2.22)	0.242	1.72 (1.17–2.52)	0.006	0.90 (0.48–1.66)	0.725
**51–60**	0.66 (0.34–1.27)	0.213	0.92 (0.71–1.21)	0.562	0.74 (0.47–1.16)	0.184	**1.81** **(1.34–2.45)**	**0.0001**	0.81 (0.46–1.43)	0.470
**61–70**	1.02 (0.52–2.01)	0.950	0.64 (0.45–0.91)	0.013	0.76 (0.51–1.13)	0.175	1.21 (0.83–1.77)	0.320	0.46 (0.19–1.15)	0.098
	0.39 (0.09–1.64)	0.201	0.84 (0.57–1.24)	0.388	0.71 (0.47–1.08)	0.112	1.22 (0.89–1.69)	0.221	1.36 (0.63–2.91)	0.434

^1^ Univariable Cox regression analysis with likelihood chi-square statistic; HR: Hazard Ratio, CI: confidence interval, bold values denote statistical significance at the *p* < 0.001 level.

## Data Availability

The data and the code used for this study are available from the corresponding author upon reasonable request. Due to the proprietary nature of the Disease Analyzer database and IQVIA’s terms of data use agreement, research data cannot be shared.
